# Long-Term Stability and Integrity of Plasmid-Based DNA Data Storage

**DOI:** 10.3390/polym10010028

**Published:** 2018-01-01

**Authors:** Hoang Hiep Nguyen, Jeho Park, Seon Joo Park, Chang-Soo Lee, Seungwoo Hwang, Yong-Beom Shin, Tai Hwan Ha, Moonil Kim

**Affiliations:** 1 Hazards Monitoring Bionano Research Center, Daejeon, 34141, Korea; nguyenhoanghiep244@gmail.com (H.H.N.); parkjeho88@gmail.com (J.P.); seonjoopark86@kribb.re.kr (S.J.P.); cslee@kribb.re.kr (C.-S.L.); ybshin@kribb.re.kr (Y.-B.S.); 2 Department of Nanobiotechnology, Korea University of Science and Technology (UST), 217 Gajeongno, Yuseong-Gu, Daejeon 34113, Korea; 3 Korean Bioinformation Center, Korea Research Institute of Bioscience and Biotechnology (KRIBB), 125 Gwahangno, Yuseong-Gu, Daejeon 34141, Korea; swhwang@kribb.re.kr; 4 Department of Pathobiology, College of Veterinary Medicine Nursing & Allied Health (CVMNAH), Tuskegee University, Tuskegee, AL 36088, USA

**Keywords:** DNA, data storage, plasmid, long-term storage, stability, integrity

## Abstract

Validation of long-term DNA stability and integrity are essential for the use of DNA in data storage applications. Because of this, we evaluated the plasmid-based DNA data storage in a manner that preserves DNA stability and integrity. A document consisting of 2046 words was encoded with DNA sequences using Perl script, and the encoded DNA sequences were synthesized for information storage. The DNA comprised a total of 22 chemically synthesized DNA fragments with 400 nucleotides each, which were incorporated into a plasmid vector. A long-term DNA stability study demonstrated that 3-year stored plasmid containing text information showed DNA stability at controlled conditions of −20 °C. The plasmid DNA under accelerated aging conditions (AAC) up to 65 °C for 20 days, which corresponds to approximately 20 years of storage at −20 °C, also exhibited no significant differences in DNA stability compared to newly produced plasmid. Also, the 3-year old plasmid stored at −20 °C and the AAC-tested plasmid stored up to 65 °C for 20 days had functional integrity and nucleotide integrity comparable to control sample, thereby allowing for retrieval of the original error-free text data. Finally, the nucleotides were sequenced, and then decoded to retrieve the original data, thereby allowing us to read the text with 100% accuracy, and amplify the DNA with a simple and quick bacterial transformation. To the best of our knowledge, this is the first report on examining the long-term stability and integrity of plasmid-based DNA data storage. Taken together, our results indicate that plasmid DNA data storage can be useful for long-term archival storage to recover the source text in a reproducible and accountable manner.

## 1. Introduction

In this digital age, developing a digital data storage strategy is very important because the storage media for information are inherently inconsistent and prone to obsolescence due to rapid and remarkable advances in information technology. Examples of digital data storage media are hard-disk drives, solid state storage, magnetic tapes, flash drives, and optical discs. Digitization of text information, most often in the form of binary code that uses only zeros and ones, allows for space reduction in repositories by reducing the high volume of hard copy outputs because printed texts are heavy and occupy shelf space. Despite the many benefits of digital data storage devices in terms of storage capacity and density, an electronic data storage device has a limited life expectancy, thus will ultimately stop functioning and subsequently lose its ability to store information just in time. There is a lifetime limit on writing even to flash memory. One of its weaknesses is that reading flash memory slightly degrades surrounding stored values, which can eventually lead to errors. Therefore, alternative media for digital storage devices with improved integrity and stability are required to overcome their susceptibility to physical damage or catastrophic loss.

Only recently has much attention been given to DNA data storage, which is a biological device to store information in a DNA sequence [1,2,3]. DNA data storage has many advantages over current digital storage media summarized as follows: (1) DNA has a great storage density and capacity; (2) DNA can store data without electricity consumption, and (3) DNA is extremely long-lasting. In 1999, DNA-based steganography was reported by Clelland et al. [4]. To hide the message, they used DNA microdot camouflage technology, in which secret messages were concealed in the human genomic DNA as a microdot and were properly secured. Further work was published in Science by the same group [5]. In their study, two types of DNA, information DNAs (iDNAs) and polyprimer key (PPK) designed to be effective in encryption, were utilized. In 2003, Wong et al. reported the concept of DNA data storage by storing information-harboring DNA in a living organism [6]. The authors inserted artificial DNA sequences into prokaryotes, in which a short verse of the children’s song “It’s a Small World” was encoded into bacteria DNA to show that bacteria could be used as a means of data storage. In 2012, Church et al. reported on a scalable DNA storage scheme in Science using the next-generation sequencing technologies based on the sequencing-by-synthesis approach, encoding an HTML version of Church’s book “Regenesis” that includes 53,426 words, 11 images in JPEG format and a JavaScript program, into DNA [7]. More recently, data storage in DNA that is more scalable for practical use was published in Nature in 2013 by Nick Goldman et al. [8]. The authors showed the potential for DNA-based high-density storage by storing five different types of files, all 154 Shakespeare sonnets in TXT format, PDF of Watson and Crick’s 1953 paper, Martin Luther King’s “I Have a Dream” speech as MP3 file, JPEG 2000 format of EMBL-EBI, and Huffman code in ASCII text, into DNA. Most recently, Grass et al. reported an interesting error-free storage of digital data by storing 83 kb of information in 4991 DNA segments, each 158 nucleotides long, which were encapsulated in silica spheres, and employing error-correcting coding scheme to correct mistakes in the data [9]. Despite such an improved scale of data storage in DNA, little study has been performed on evaluating DNA stability in terms of quality and DNA integrity in the field of DNA data storage.

In this study, for the use of DNA in data storage applications, assessment of long-term DNA stability and integrity are primarily required. For this, herein, we performed long-term stability analyses with plasmid DNA stored at −20 °C for 3 years as well as under accelerated aging conditions (AAC) up to 65 °C for 20 days, which corresponds to approximately 20 years of storage at −20 °C, for the monitoring of stability and integrity of plasmid DNA for data storage.

## 2. Materials and Methods

### 2.1. Perl Script

A script was written in the Perl programming language. In the encoding mode, the script converts each character in the text document into a base quadruplet based on the 4-base code system, which was implemented in the script as a hash-based lookup table. In the decoding mode, the script first removes adapter sequences from each sequence fragment, then converts each of the four residues into the corresponding character using the reverse of the hash table, and finally concatenates the sentence fragments into a text document.

### 2.2. Cloning of Information DNA

In order to clone information DNA, a total of 8184 nucleotides that represent the preamble of the UDHR were divided into 22 partial length fragments with 400 nucleotides each, and then, these fragments were obtained by chemical synthesis. The 400 nucleotides were PCR-amplified with 5′ and 3′ primers (Table 1), and their 5′ and 3′ termini were designed to harbor the *Eco*RI restriction enzyme cleavage sites. The PCR products were then purified with a DNA purification kit (Qiagen, Valencia, CA, USA), and digested with the corresponding restriction enzyme. The DNA fragment was ligated into the pBHA cloning vector (Bioneer Inc., Daejeon, Korea) to generate the plasmid DNA for data storage, and verified via DNA sequencing.

### 2.3. Storage of Plasmid DNA

The pBHA plasmid vector containing a 0.4 kb information insert was used for all experiments. Each plasmid DNA (final concentration, 1 ng/uL) dissolved in TE buffer (10 mM Tris–Cl, pH 7.5; 1 mM EDTA) was stored in a 0.5-mL tube at −20 °C for 3-year period from April 2014 through March 2017, and maintained at 23, 37, 45 and 65 °C for 20 days for accelerated aging conditions (AAC) test. Newly produced plasmid DNA used as a control was extracted from newly transformed *E. coli* with the same plasmid DNA using a plasmid miniprep kit (Qiagen, Valencia, CA, USA).

### 2.4. Agarose Gel Electrophoresis (AGE) Analysis

The molecular weight or DNA quality of the applied plasmid DNA was examined by running 10 ng of the plasmid DNA per lane directly on a 1.2% agarose gel stained with ethidium bromide (EtBr). For quantification of DNA, densitometric measurement was performed using GS-900™ Densitometer (Bio-Rad Laboratories, Inc., Headquarters, Hercules, CA, USA).

### 2.5. Bacterial Transformation

The recombinant plasmid harboring text information was transformed into *Escherichia coli* DH5α cells by calcium chloride (CaCl_2_) heat shock method in order to isolate the plasmid DNA. Briefly, CaCl_2_-treated *E. coli* cells were incubated with 10 ng of DNA on ice for 30 minutes to enable the plasmid DNA to move toward the cell membrane, and then heated to 42 °C for 90 seconds to expedite the plasmid to enter the bacterial cells. The transformed cells were grown at 37 °C with shaking in culture tubes containing 5 mL of LB/ampicillin media (100 μg/mL of ampicillin) to an OD_600_ of 0.6. The resultant plasmid DNA was then extracted using a plasmid miniprep kit (Qiagen, Valencia, CA, USA).

## 3. Results and Discussion

### 3.1. Encoding

**Scheme 1:**
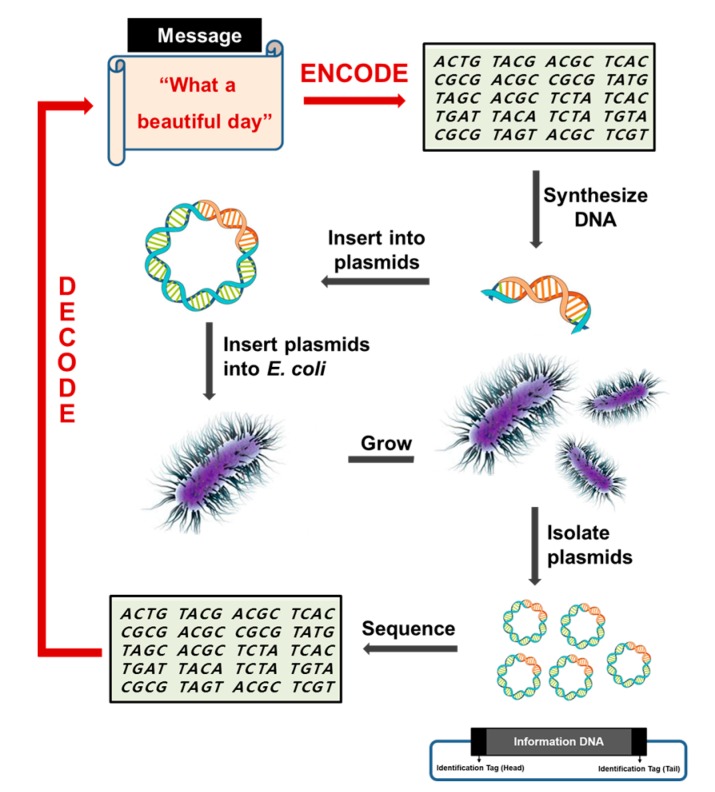
Schematic diagram showing DNA data storage. A text document is encoded into DNA, and then the cognitive DNA was chemically synthesized. Following DNA sequencing for data retrieval, the DNA sequences are ultimately decoded into the original document. Information DNA comprising 8184 nucleotides was split into 22 separate DNA fragments with 400 nucleotides each. Each fragment included 14-bp flanking regions as sites for sequencing primers or as identification tags to combine the DNA segments in the proper order, and was inserted into a cloning vector using the *Eco*RI enzyme. The DNA tag is shown in the black box, and the gray box represents the information DNA site. Not drawn to scale.

**Figure 1:**
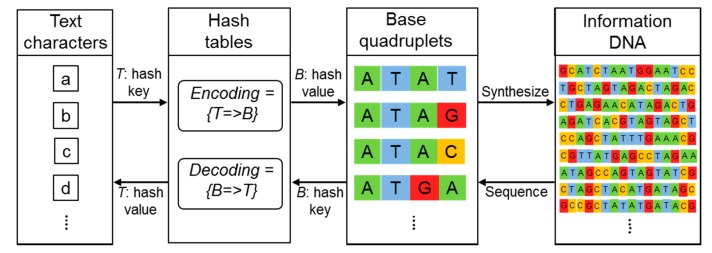
Processes of encoding and decoding. These processes were implemented in a Perl script. The schematic thus contains some Perl-style pseudo-codes. See Materials and Methods for details.

In biology, the main function of DNA in living organisms is to store genetic information as genes. In this regard, DNA is characterized by two properties essential for its basic function. First, DNA must be chemically and physically stable to minimize the risk of damage. Second, DNA must be able to produce identical copies of the information. With these structural and functional properties of DNA, the digital information in a computer can be stably encoded in and decoded from DNA (see Scheme 1). The first step to develop DNA data storage is to prepare for lookup table for encoding the information into nucleotide sequence. To fully represent the character set in usual English documents, we adopted a straightforward 4-base coding scheme that represents a character with a nucleotide base quadruplet. The reason why a 4-bit code was used as a character-encoding scheme in this work is so that the 128 ASCII (American Standard Code for Information Interchange) characters could be represented including the upper- and lower-case English alphabet, numbers from 0 to 9, punctuation marks, and symbols while a 3-bit code can only encode 64 distinct letters, since 4-bit code can produce up to 256 (4^4^) different base quadruplets. For encoding, a script written in Perl (Practical Extraction and Report Language), which is the best-known programming language for text processing and can be run on any operating systems with Perl interpreter installation, was used to convert the text data into a DNA sequence (Figure 1).

**Figure 2:**
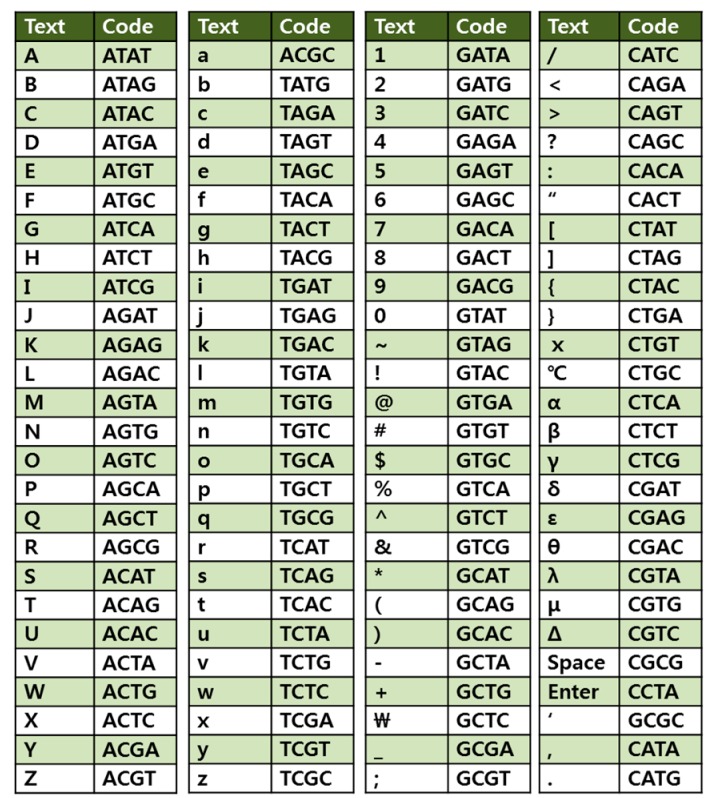
Encoding lookup table. Four-base code was used to represent each character. With a four-base code, 256 different characters including upper case A through Z, lower case a through z, numbers 0 through 9, punctuation marks and symbols on the keyboard are encoded.

To encode each character with a base quadruplet based on the 4-base code system, we designed a lookup table shown in Figure 2. With a four-base code, 256 different characters including upper case A through Z, lower case a through z, numbers 0 through 9, punctuation marks and symbols on the keyboard are encoded. Using the Perl script-based encoding program, the preamble of the “Universal Declaration of Human Rights” was encoded into 8,184 nucleotides, which were divided into a total of 22 DNA fragments. Each DNA fragment was flanked by head and tail tags with different 14-bp DNA adapter sequences that serve as sequencing primers or identification tags for subsequent data retrieval to assemble the DNA fragments in the right order as a continuous sequence block.

### 3.2. Data Storage in Plasmid DNA

The DNA fragments were artificially synthesized with the ligase chain reaction (LCR) method through a gene synthesis service. The synthesized DNA fragments were amplified with the 5′ and the 3′ primers (Table 1), respectively, with the polymerase chain reaction (PCR). Their 5′ and 3′ termini were designed to include the *Eco*RI restriction enzyme site. After digestion with the indicated enzyme, each fragment was inserted into the pBHA cloning vector using the *Eco*RI cleavage site to produce plasmids containing information DNA. The information DNA fragments were all verified by DNA sequencing, and transformed into *E. coli* DH5α for multiplication of the plasmid DNA. The transformed cells could potentially multiply the plasmid DNA countless times through plasmid replication in cells based on the plasmid copy number and by the cell division of bacteria, thereby requiring no PCR amplification to obtain the desired numbers of copies. In cells, mutations may occur due to unrepaired damage to DNA and errors during the replication process. Considering that bacterial cells have an elaborate DNA repair system of their own in response to mutations and errors found in genomic DNA as well as plasmid DNA during the replication process, it can be assumed that plasmid DNA is likely to ensure its integrity, when bacterial cells are used for transforming plasmid DNA for the purposes of storage and amplification.

### 3.3. Analysis of Long-Term Stability and Integrity of Plasmid DNA

**Figure 3:**
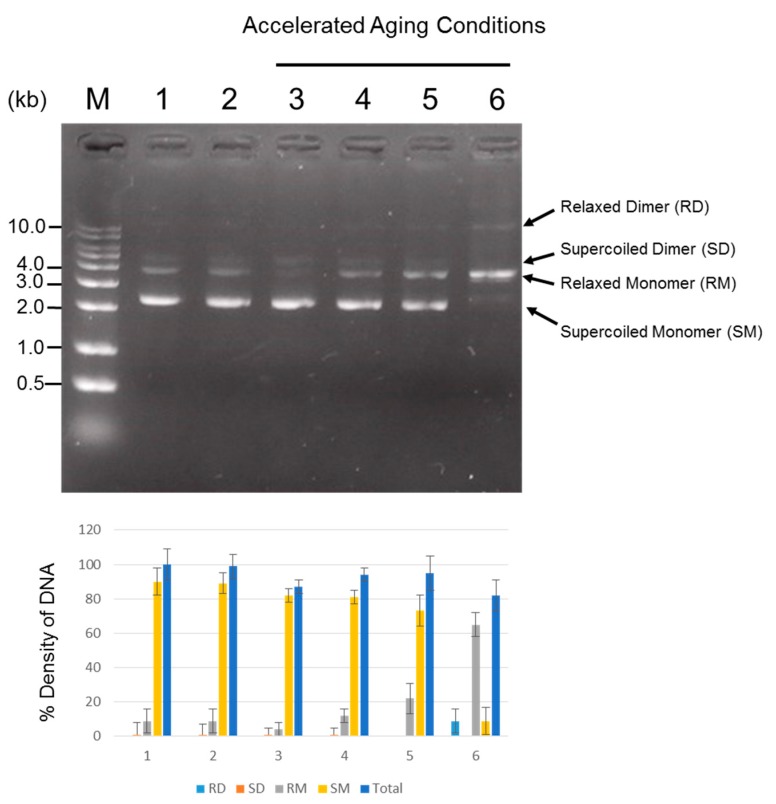
Analysis of plasmid DNA quality and quantity. One hundred ng of plasmid DNA was loaded to agarose gel wells and allowed to run. AGE was used to monitor the degree of DNA degradation or the conversion of the supercoiled form to the relaxed form. For quantification of DNA, densitometry was performed. The density of newly produced plasmid DNA as a control was set at 100%, and the band densities relative to the control are shown. The experiments were repeated twice in triplicate; bar, SE. The plasmid DNA under accelerated aging conditions (AAC) to 23 °C, 37 °C, 45 °C and 65 °C temperatures for 20 days corresponds to approximately 13 months, 34 months, 5 years and 20 years of storage at −20 °C, respectively. Newly produced plasmid DNA was used as a control. A *p*-value < 0.05 was considered statistically significant for confidence. M: DNA ladder; Lane 1: Newly produced plasmid DNA (Control); Lane 2: 3-year old plasmid DNA stored at −20 °C; Lane 3–6 (AAC test): Plasmid DNA stored at 23 °C, 37 °C, 45 °C and 65 °C for 20 days.

Long-term stability of DNA is of great importance to DNA-based data storage. Thus, it is necessary to evaluate plasmid DNA stability and integrity during long-term storage. For this aim, plasmid containing text information was produced for DNA data storage study started in April 2014, and was subsequently stored at −20 °C for 3-year period. Also, accelerated aging conditions (AAC) to reduce the test time to the maximum extent for the measurement of the plasmid stability and integrity was tested on the plasmid DNA. Agarose gel electrophoresis (AGE) and transformation efficiency analyses were performed to examine the long-term stability and integrity. Also, densitometry analysis was performed to quantify the amount of DNA. Using AGE analysis, DNA stability can be monitored by comparing the degree of visible DNA degradation represented by the smear appearing on the gel. For this, ten ng of each sample of plasmid DNA stored at −20 °C for 3 years, stored at 23, 37, 45 and 65 °C for 20 days, and newly produced plasmid control were loaded on a 1.2% agarose gel, and DNA quality was electrophoretically examined. As shown in Figure 3, AGE data showed that in all samples, no smear was detected without linearization or degradation of the plasmid on the gel, indicating protection of the applied plasmid DNA from degradation under our experimental conditions. In general, uncut plasmids contain at least two topologically different states of DNA, corresponding to predominant supercoiled forms and relaxed forms. In control sample (lane 2), two prominent bands were visible, and a similar banding pattern was seen in other lanes (lane 3–6). Interestingly, however, the predominant supercoiled species of plasmid disappeared and an additional band of 10 kb was faintly visible in the lane of AAC-tested plasmid stored at 65 °C for 20 days. This could be a thermal effect, resulting in the formation of more relaxed structures, implying that the harsh aging conditions (65 °C for 20 days) could induce the conversion of the supercoiled form to the relaxed state. Our findings indicated that plasmid containing information DNA stored at controlled conditions of −20 °C could possess DNA stability at least for 3 years, and the plasmid could be protected from degradation even under elevated conditions of temperature up to 65 °C for 20 days, which is equivalent to approximately 20 years of DNA storage at −20 °C.

**Figure 4:**
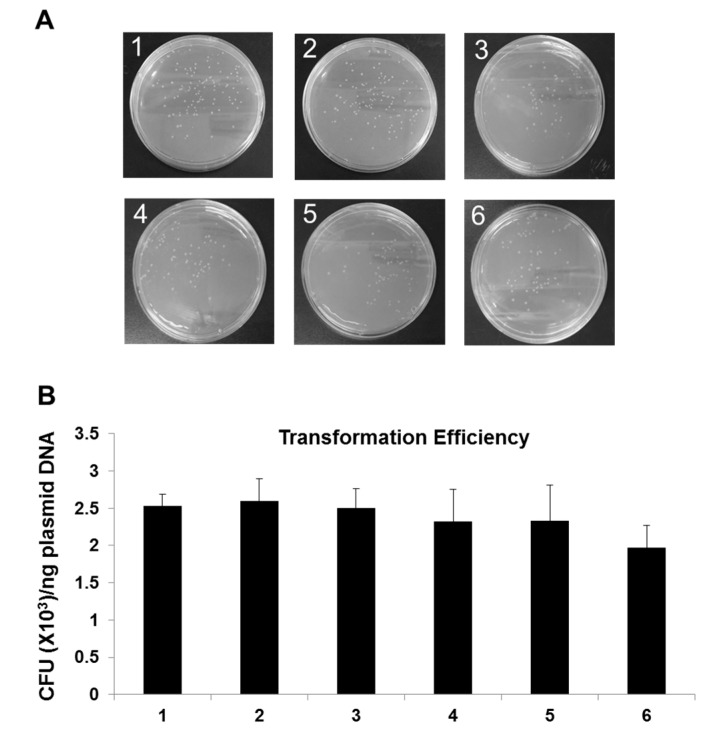
Comparison of colony formation of *E. coli* transformed with plasmid containing text information. (**A**) Pictures of LB plates with ampicillin after an overnight incubation to visualize transformation efficiency. The competent *E. coli* DH5α transformed cells that have been diluted 1:50 were spread on ampicillin plates. (**B**) Comparison of transformation efficiency. One ng of each plasmid DNA sample was used for transformation of *E. coli*. Newly produced plasmid DNA was used as a control. A *p*-value < 0.05 was considered statistically significant for confidence. 1: Newly produced plasmid DNA (Control); 2: 3-year old plasmid DNA stored at −20 °C; 3–6 (AAC test): Plasmid DNA stored at 23 °C, 37 °C, 45 °C and 65 °C for 20 days.

After the measurement of the quality of plasmid harboring information DNA, the functional integrity of the plasmid DNA was examined by transformation in *E. coli* and antibiotic selection with ampicillin, since the pBHA has a gene for ampicillin resistance. In order to test the impact of long-term storage on its functionality, the plasmid DNA was transformed in *E. coli* and the functional integrity was calculated based on the transformation efficiency as CFU (colony-forming unit) numbers of colonies/ng transformed DNA. As shown in Figure 4, the transformation efficiency for newly produced plasmid control, plasmid DNA stored at −20 °C for 3 years, plasmid DNA stored at 23 °C, 37 °C, 45 °C and 65 °C for 20 days was 2.5 CFU (×10^3^)/ng plasmid DNA, 2.6 CFU (×10^3^)/ng plasmid DNA, 2.3 CFU (×10^3^)/ng plasmid DNA, 2.3 CFU (×10^3^)/ng plasmid DNA, and 2.0 CFU (×10^3^)/ng plasmid DNA, respectively. In all tested samples except for AAC-tested plasmid at 65 °C for 20 days, no significant difference in transformation efficiency was found, showing colony counts comparable to control sample (*p* < 0.05). A slight reduction in transformation efficiency in response to AAC-tested plasmid at −20 °C for 20 days was observed, however, representing no statistically significant difference. Negative control was simultaneously tested and no colony was detectable indicating no bacterial contamination (Data not shown). 

Functionality tests in *E. coli* revealed that all plasmid DNA samples were biologically active, irrespective of storage conditions used here. In addition, the nucleotide integrity of the plasmid carrying text information was confirmed by DNA sequencing. Out of 22 information DNA fragments, randomly selected DNA fragments #1 were subject to sequencing analysis. A single colony from each transformation was carefully picked up for amplification and sequencing. For information DNA fragment #1 as a representative sample, DNA sequences of newly produced plasmid control, 3-year old plasmid DNA stored at −20 °C, and AAC-tested plasmid DNA stored at 23 °C, 37 °C, 45 °C and 65 °C for 20 days were 100% homologous to the original sequence without mutation, insertion and deletion (Data not shown). These results indicated that the nucleotide integrity was ensured on the plasmid DNA examined under the realistic long-term storage conditions as well as the accelerated testing conditions used in this study, thereby allowing for retrieval of the original error-free text data.

Murakami reported that plasmid DNA should be maintained in TE (Tris–EDTA) buffer at −20 °C for longer-term storage [10]. Given that DNA is pH sensitive, Tris as a pH buffer plays an important role in maintaining a stable pH, despite influences that might otherwise change the pH. Also, it should be noted that EDTA chelates divalent ions, rendering them inactive, which are common in nucleases. Therefore, the protective role of TE buffer in being resistant to DNA degradation can pay off during long-term storage. In this context, our findings demonstrated that minimal protection made by TE buffer at controlled conditions of −20 °C was sufficient to prevent the applied plasmid DNA from degradation, leading to its functional integrity, at least under our experimental settings.

### 3.4. Decoding

The first step of decoding is DNA sequencing in which there could be a possible problem caused by weak signals or background noise, and multiple peaks with the same height might appear on the sequence map. To solve these problems, bidirectional sequencing, in which ambiguous sequences in one strand of the double helix DNA sequences can be easily resolved using the sequence of the complementary strand, was used with sequence-reading done inward from the two ends of each DNA fragment. By combining the forward and reverse sequencing reactions, an error-free rate for each of the 22 sequencing reactions was achieved. Additionally, it is note-worthy that time and reagent consumption can be halved by the use of the bidirectional sequencing method.

Each plasmid contained 400-bp DNA sequences including 14-bp flanking sequences as identification markers attached to the ends of the fragments to restore the DNA segments in the proper order. To properly order the 22 sequenced shredded DNA fragments, Perl script-based assembly program was produced to recognize the 14-bp adapter sequences from each fragment. The Perl script allowed for the identification of the entire data sequence by combining the divided DNA fragments in one stretch. The randomly scattered 22 DNA fragments were rearranged into a large chunk of DNA. Finally, in order to retrieve data stored in the DNA, the DNA was decoded using Perl script-based decoding program by converting a base quadruplet into its corresponding character according to the lookup table-based Perl script. After decoding, the document identity between the original text and the retrieved text was examined with WinMerge 2.14.0 program, which can compare both folders and files (http:winmerge.org). The two documents are perfectly identical, indicating that DNA can be potentially applicable as an excellent storage media to recover the source text.

## 4. Conclusions

To the best of our knowledge, this is the first report on examining the long-term stability of plasmid-based DNA data storage. The aim of this article was to evaluate plasmid-based DNA data storage in a manner that preserves DNA stability and integrity. In this study, the documented message, the preamble of the “Universal Declaration of Human Rights”, was reliably encoded into synthetic DNA through the 4-base DNA code system. The plasmid DNA containing text data was stored at −20 °C for 3 years or stored under accelerated aging conditions (AAC) up to 65 °C for 20 days, which corresponds to approximately 20 years of storage at −20 °C, for the monitoring of the long-term DNA stability and integrity. The plasmid DNA tested under the realistic long-term storage conditions as well as the accelerated testing conditions showed DNA stability, functional integrity, and nucleotide integrity during long-term storage, thereby allowing for retrieval of the original error-free text data. Therefore, our results indicated that plasmid-based DNA data storage could be beneficial as a long-term storage medium in a reproducible and accountable manner. This study also revealed that an undesired DNA base could be successfully corrected by simply running the overlap extension PCR reaction as a strategy for repair of erroneous DNA. The resultant information DNA was sequenced and subsequently decoded by converting DNA code into its corresponding character according to the lookup table-based Perl script. Following decoding, the original document was successfully retrieved with 100% accuracy, allowing us to amplify the information DNA with a simple and quick bacterial transformation. Although this study demonstrated a minuscule amount of document data and a limited encoding scheme that works for only English text-based data, at least our data provided strong indication that plasmid-based DNA data storage could be a useful strategy for long-term archival storage. There are five primary aspects one needs to consider when developing digital data storage technology, namely, accuracy, density, longevity, cost, and stability. In the past decade, the concept of DNA as a storage material has been proven by researchers. Despite potential benefits of the use of plasmid DNA as a long-lasting storage medium, many issues such as encoding and decoding speed, cost-effectiveness, mutations under extreme conditions, and so on still remain to be overcome before the practical application of plasmid-based DNA data storage can be implemented [11,12,13]. Regarding cost issues, rapid advances in instrumentation technology have led to significant reductions in costs for DNA synthesis, as the cost of DNA synthesis has dropped 5-fold annually, while the cost of electronic storage media has only dropped 1.6-fold per year. Moreover, it is believed that innovative technology such as next-generation sequencing (NGS) instrumentation will enable DNA data storage to reach cost-effectiveness within a few decades.

## Figures and Tables

**Table 1:** A list of primer sequences used for information DNA cloning.

Fragments	Forward primer	Reverse primer
1	5′-GAATTCACTCATCCCAACTG-3′	5′-GAATTCAGATTGCCGAGAAT-3′
2	5′-GAATTCACGGAGCGACATGA-3′	5′-GAATTCACAGCTGTTGATAA-3′
3	5′-GAATTCCTAGCAGTTCTAAT-3′	5′-GAATTCGACGATGGCAAAAG-3′
4	5′-GAATTCGTAAAAGCCTCCAA-3′	5′-GAATTCGTATGATCAATCTC-3′
5	5′-GAATTCCTATCGGCACAGAA-3′	5′-GAATTCCGGCGTCGTGTCAC-3′
6	5′-GAATTCATGGGTAGCGGACT-3′	5′-GAATTCTGTGGTTGACCAAA-3′
7	5′-GAATTCATTCGCTAGGGGAC-3′	5′-GAATTCACGCCGCAGGACCT-3′
8	5′-GAATTCACATCACTTTGTAT-3′	5′-GAATTCTGGGCTGGTTGCAC-3′
9	5′-GAATTCAGTGGGGCCAGGCA-3′	5′-GAATTCAACCAGCTGAGTCT-3′
10	5′-GAATTCCCTGTGTTAGCTCG-3′	5′-GAATTCGTCATCCCTAGCCT-3′
11	5′-GAATTCAGCTCTTTAAACAT-3′	5′-GAATTCGACGCCCCCAGCCT-3′
12	5′-GAATTCGAACGGTCGAGAAG-3′	5′-GAATTCCGAGGTACTATGAG-3′
13	5′-GAATTCGGTACCAACTTACT-3′	5′-GAATTCCAAGCAATAGCCTG-3′
14	5′-GAATTCAAGCTGTACTATTT-3′	5′-GAATTCGCGCTCCCCCCCTG-3′
15	5′-GAATTCTGATGGTCTCTTCT-3′	5′-GAATTCGTTGAGTCATCAGA-3′
16	5′-GAATTCTCGCAAGGGTCGTG-3′	5′-GAATTCGAAGGAACCGACTT-3′
17	5′-GAATTCAATGGTTAAAAATC-3′	5′-GAATTCCACAGTGAGCCTTT-3′
18	5′-GAATTCCAGACTGGAGCGCC-3′	5′-GAATTCCGAGCCGCTAGATG-3′
19	5′-GAATTCCGTCTCGAATGCTC-3′	5′-GAATTCTGACAAAGGGGACC-3′
20	5′-GAATTCTTCCGGATAAATTC-3′	5′-GAATTCTGAATGAGGGGTAT-3′
21	5′-GAATTCCTAGCTTGCGAAGT-3′	5′-GAATTCATATACCAATGTAG-3′
22	5′-GAATTCGAATCCGACCTAGA-3′	5′-GAATTCTTTAAGTGCCCTCT-3′
